# Clinical Validation of a Proteomic Biomarker Threshold for Increased Risk of Spontaneous Preterm Birth and Associated Clinical Outcomes: A Replication Study

**DOI:** 10.3390/jcm10215088

**Published:** 2021-10-29

**Authors:** Julja Burchard, Ashoka D. Polpitiya, Angela C. Fox, Todd L. Randolph, Tracey C. Fleischer, Max T. Dufford, Thomas J. Garite, Gregory C. Critchfield, J. Jay Boniface, George R. Saade, Paul E. Kearney

**Affiliations:** 1 Sera Prognostics, Incorporated, Salt Lake City, UT 84109, USA; jburchard@seraprognostics.com (J.B.); ashoka@seraprognostics.com (A.D.P.); afox@seraprognostics.com (A.C.F.); trandolph@seraprognostics.com (T.L.R.); mdufford@seraprognostics.com (M.T.D.); tgarite@seraprognostics.com (T.J.G.); gcritchfield@seraprognostics.com (G.C.C.); jboniface@seraprognostics.com (J.J.B.); pkearney@data-incites.com (P.E.K.); 2 Department of Obstetrics & Gynecology, University of Texas Medical Branch, Galveston, TX 77555, USA; gsaade@utmb.edu

**Keywords:** preterm birth, IBP4, SHBG, biomarkers

## Abstract

Preterm births are the leading cause of neonatal death in the United States. Previously, a spontaneous preterm birth (sPTB) predictor based on the ratio of two proteins, IBP4/SHBG, was validated as a predictor of sPTB in the Proteomic Assessment of Preterm Risk (PAPR) study. In particular, a proteomic biomarker threshold of −1.37, corresponding to a ~two-fold increase or ~15% risk of sPTB, significantly stratified earlier deliveries. Guidelines for molecular tests advise replication in a second independent study. Here we tested whether the significant association between proteomic biomarker scores above the threshold and sPTB, and associated adverse outcomes, was replicated in a second independent study, the Multicenter Assessment of a Spontaneous Preterm Birth Risk Predictor (TREETOP). The threshold significantly stratified subjects in PAPR and TREETOP for sPTB (*p* = 0.041, *p* = 0.041, respectively). Application of the threshold in a Kaplan–Meier analysis demonstrated significant stratification in each study, respectively, for gestational age at birth (*p* < 001, *p* = 0.0016) and rate of hospital discharge for both neonate (*p* < 0.001, *p* = 0.005) and mother (*p* < 0.001, *p* < 0.001). Above the threshold, severe neonatal morbidity/mortality and mortality alone were 2.2 (*p* = 0.0083,) and 7.4-fold higher (*p* = 0.018), respectively, in both studies combined. Thus, higher predictor scores were associated with multiple adverse pregnancy outcomes.

## 1. Introduction

Preterm birth (PTB), including both spontaneous (sPTB) and medically indicated (miPTB) birth before 37 weeks gestation, occurs in approximately 10% of all births in the US and is a leading cause of neonatal morbidities, mortality and long-term health consequences worldwide [1,2]. PTB and associated morbidities, such as respiratory distress, can require extended stays and care in neonatal intensive care nurseries, along with increased costs [3]. The application of existing interventions such as progestogens and systems of care coordination, and the effective development of new interventions depend on screening tools to identify pregnancies at risk. Clinical markers associated with an increased risk of sPTB are present in only a minority of pregnancies, limiting their overall utility. A history of previous sPTB is a traditional predictor of recurrent sPTB but applies to only approximately 4% of all pregnancies and 11% of all sPTBs [4,5]. Similarly, a short cervical length measured by transvaginal ultrasound is a predictor of sPTB, but accounts for only an additional 2% of all pregnancies and 6% of all sPTBs [6,7].

In accordance with the National Academy of Medicine’s guidelines [8] for the rigorous development of multi-biomarker tests, clinical validity is ideally replicated in a second study, independent from the one in which the test was originally developed. Additionally, it is desirable for a test to have a prespecified threshold to risk-stratify subjects so that clinicians can easily interpret and act upon test results. In the Proteomic Assessment of Preterm Risk (PAPR) study, Saade et al. reported the development and clinical validation of a serum test for sPTB prediction that utilizes the proteomic biomarker of insulin-like growth factor binding protein-4 (IBP4) and sex hormone binding globulin (SHBG) [9]. These two proteins, used in combination, were found to be the most predictive pair of biomarkers amongst hundreds of proteins screened during a systems biology approach in the PAPR study. IBP4 is expressed in syncytiotrophoblasts and negatively regulates insulin-like growth factors [10], key regulators of placental development [11]. SHBG, primarily secreted by the liver, is also placentally expressed [12], and circulating SHBG levels increase ~5-fold during pregnancy [13]. SHBG regulates the bioavailability of sex hormones, is associated with diabetes and insulin resistance [14] and is negatively regulated by proinflammatory cytokines [15] implicated in etiologies of PTB.

In the subsequent validation of IBP4/SHBG, in addition to demonstrating a statistically significant area under the receiver operating characteristic (AUC) curve for predicting preterm birth, the study reported that subjects with a proteomic biomarker score at or above −1.37 delivered earlier than those with lower proteomic biomarker scores [9]. The study showed that subjects at or above a proteomic biomarker threshold of −1.37, corresponding to a risk probability of ~15%, are at approximately 2-fold or greater increased risk of sPTB as compared to the average risk of singleton pregnancies in the United States.

The primary objective of the current analysis was to demonstrate that significance of proteomic biomarker thresholds is replicated across independent studies. Of particular importance was extending the work of Saade and colleagues [9] by demonstrating that the risk of sPTB is significantly elevated at the proteomic biomarker threshold of −1.37 in two additional cohorts. First, an expanded, but partially overlapping, cohort of subjects from the PAPR study was utilized to verify that sPTB remains significantly elevated in patients with a score above the threshold. Second, we conducted a validation of the threshold in a large and completely independent cohort, the Multicenter Assessment of a Spontaneous Preterm Birth Risk Predictor (TREETOP) study (NCT02787213) [16]. In clinical practice, risk probabilities are utilized rather than proteomic biomarker thresholds. We present performance results for the proteomic biomarker threshold of −1.37 which has been shown to correspond to the risk probability of 15% [9].

The second objective was to assess whether the threshold can identify elevated risk of all PTB (sPTB and medically indicated PTB) and pregnancy complications associated with prematurity: increased lengths of maternal and neonatal hospital stay and severe neonatal morbidity and mortality. Such results are particularly important as not all premature pregnancies result in adverse outcomes, and so, demonstrating that the proteomic biomarker threshold also stratifies pregnancies by adverse neonatal and maternal outcomes adds direct evidence to the potential clinical utility of the proteomic biomarker predictor. We note that a previous exploration of the proteomic biomarker on the TREETOP cohort did not address threshold validity [16]; that is, the work did not validate a pre-specified threshold, nor did it assess the ability of the proteomic biomarker to stratify patients at any specific predictor score threshold for any outcome.

## 2. Materials and Methods

### 2.1. PAPR and TREETOP Subpopulation Selection

Subpopulations of the PAPR (NCT01371019) and TREETOP (NCT02787213) studies were selected to conduct this prospective-retrospective cohort analysis as described below. We refer to these two subpopulations as the verification and validation cohorts, respectively, in accordance with the National Academy of Medicine’s Guidelines for test development [8].

The proteomic biomarker and a specific threshold were developed and fully defined in the original study [9]. The verification cohort for the current analysis was the subpopulation of PAPR consisting of all subjects (*n* = 549) meeting the following criteria: did not receive progesterone on or after 14 weeks gestation, underwent sample collection in the validated gestational age window (19^1/7^–20^6/7^ weeks) [9] and gave consent for future research use of their deidentified samples and data. Of the 549 subjects in this verification phase, only 32 were previously used for discovery of the classifier in the original study [9].

The validation cohort for the current analysis was the subpopulation of TREETOP consisting of a randomly selected subset (*n* = 847), or 34% of all subjects who underwent sample collection in the validated gestational age window (19^1/7^–20^6/7^ weeks) [16]. TREETOP is an observational study of pregnant women who did not receive progesterone on or after 14 weeks gestation and included iatrogenic and spontaneous PTBs, term births and co-morbid conditions. The TREETOP subjects that were not selected for the current analysis remain blinded for future studies. Importantly, the validation cohort is fully independent of the original training and verification cohorts with no subjects in common.

As a measure of neonatal outcome and accounting for major morbidity in the prematurely delivered newborns, we adapted a previously reported Neonatal Morbidity and Mortality Index (NMI, range 0 to 4) [6]. For a surviving neonate, the reported index can be 0, 1, 2 or 3 based on newborn intensive care unit length of stay or associated diagnoses, whichever is higher. For the NICU length of stay, 1–4 days stay gives a score of 1, 5–20 days a score of 2 and >20 days a score of 3. For the associated diagnoses, a unit is added for each diagnosis of respiratory distress syndrome, bronchopulmonary dysplasia, intraventricular hemorrhage grade III or IV, necrotizing enterocolitis, periventricular leukomalacia or proven severe sepsis, up to a maximum of 3. A score of 4 is assigned to perinatal mortality. Since we did not record whether the neonate was admitted to the NICU in PAPR, total length of newborn hospital stay was used in place of NICU length of stay to calculate NMI. Because all neonates had at least one day of hospital stay, our modified scale does not start at 0 as in the PREGNANT trial [6], but with an NMI of 1. Data collection through 28 days of life allowed for confirmation of all conditions contributing to NMI.

### 2.2. Sample Analysis

All subject samples from PAPR and TREETOP were analyzed in a certified lab according to standard operating protocol using a methodology previously validated and documented [17]. Briefly, serum samples were depleted of the top fourteen most abundant proteins (MARS14, Agilent Technologies, Inc., Santa Clara, CA, USA), reduced, alkylated, and digested with trypsin [9,17]. Following digest, the samples were spiked with stable isotope standard (SIS) peptides for proteins of interest, desalted and analyzed by reversed-phase liquid chromatography multiple reaction monitoring mass spectrometry [9,17]. The extended PAPR cohort samples were analyzed retrospectively for this study. Importantly, the TREETOP samples were analyzed prospectively, as they were collected, as would be the case in actual clinical use. Relative levels of IBP4 and SHBG were expressed as response ratios (RR) of the peak area for the endogenous peptide divided by the peak area of the SIS peptide [9,17]. The IBP4/SHBG proteomic biomarker was calculated as: ln(RR_IBP4_/RR_SHBG_).

### 2.3. Data Analysis Methodology

Clinical and demographic variables were compared between the two study cohorts, with significance (*p* < 0.05) determined by Fisher’s exact test for categorical variables and Welch’s T-test for continuous variables. Variables including pre-pregnancy weight, race, ethnicity, and maternal education were self-reported, whereas obstetric history, delivery, and neonatal outcomes were collected from medical records review. Gestational age was based on best obstetrical estimate with LMP confirmed by ultrasound, with first trimester ultrasound confirmation required in TREETOP [18].

All analyses were pre-specified in a study protocol, including the fixed sequence of proteomic biomarker thresholds per outcome and the hypothesis test. Outcomes included sPTB and overall PTB, gestational age at birth (GAB), neonatal and maternal lengths of hospital stay and NMI. The hypothesis test was prespecified as a regression test with covariates and was utilized for both categorical and continuous outcomes. A binary variable for threshold (1 for proteomic biomarker scores greater than or equal to the threshold and 0 for values below the threshold) was tested as a predictor of adverse outcomes in regression with body mass index (BMI) as a covariate, indicated by the reported influence of BMI stratification reported by Saade and colleagues [9]. The significance of contribution of the binary predictor to the regression test was used as the measure of significance of prediction. Missing BMI values were found in 12/847 TREETOP subjects and 10/549 PAPR subjects; these subjects were dropped from the regression analyses. The protocol prespecified a hypothesis of increased PTB risk above the proteomic biomarker threshold. The regression analysis tested for one-sided significance of the binary threshold variable in prediction of an adverse outcome at alpha of 0.05. The same test of significance was used in the verification (PAPR) and validation (TREETOP) cohorts to avoid bias. To correct for multiple testing, outcomes were tested in a prespecified order in a fixed sequence approach, with alpha of 0.05. An independent statistician (see Acknowledgements) conducted pre-specified fixed sequence hypotheses testing. In exploratory analyses, statistical significance for Kaplan–Meier analysis was assessed by the log-rank statistic. To obtain even more robust estimates of threshold performance, the verification (PAPR) and validation (TREETOP) subjects were also combined for post-hoc analyses. The sensitivity and specificity of the threshold to be validated were compared to the sensitivity and specificity of the proteomic biomarker at the threshold of maximum accuracy [9] using McNemar’s test. As a descriptive statistic, the fold change in the occurrence of outcomes above and below the threshold was calculated as the ratio of the rate of the outcome amongst subjects with proteomic biomarker scores at or above the threshold to the rate amongst those with proteomic biomarker scores below the threshold. All analyses were performed in R version 3.5.1 [19] using packages data.table [20], demoGraphic [21], and survival [22,23].

## 3. Results

The characteristics of the verification (PAPR) and validation (TREETOP) subpopulations included in this analysis are summarized in Table 1. Compared to subjects in TREETOP, women in PAPR were younger, with a higher BMI, less educated, less likely to identify as non-white, and more likely to have had a prior sPTB. They also were more likely to have delivered preterm in the index pregnancy (Table 1). Other clinical variables were not significantly different between the two cohorts.

All outcomes were significantly predicted in TREETOP by at least the first threshold specified in the fixed sequence. Of particular interest, the proteomic biomarker threshold of −1.37 highlighted by Saade et al. [9] was statistically significant for increased sPTB in PAPR and in TREETOP (each, coincidentally, at *p*-value 0.041). Similarly, subjects at or above the threshold delivered earlier than those below the threshold in both studies by log-rank test (PAPR: *p*-value < 0.001; TREETOP: *p*-value 0.0016). In the combined cohort, preterm birth was significantly elevated in frequency at and above the threshold (sPTB, *p* = 0.0067, 1.8×; miPTB, *p* = 0.0052, 2.1×; PTB < 37 weeks gestation, *p* < 0.001, 1.9×; PTB < 35, *p* = 0.011, 2.1×; PTB < 32, *p* = 0.0064, 4.3×). The previously reported sensitivity and specificity measures for sPTB were 75% and 74%, respectively, at the proteomic biomarker threshold of maximum accuracy [9]. Sensitivity and specificity at the clinically used risk probability threshold of 15% (corresponding to a proteomic biomarker threshold of −1.37) specified in this study, were not statistically different (McNemar’s test, *p* = 0.48), with significant prediction and elevation of sPTB for scores at and above the threshold (*p* = 0.019, 3.2×).

**Figure 1:**
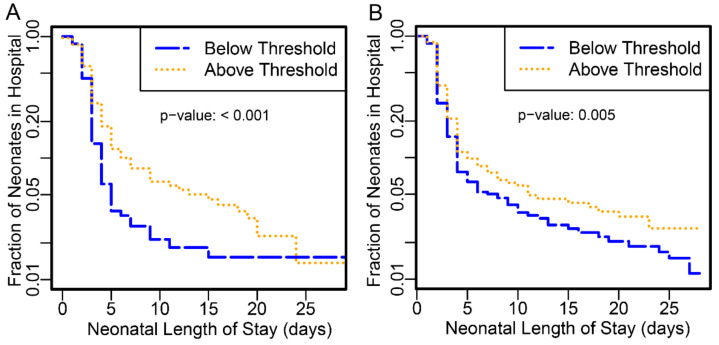
Kaplan–Meier curve of neonatal length of stay in days for all neonates. The fraction of neonates remaining in the hospital is plotted as a function of the length of hospital stay in days. Neonates without recorded hospital stays were omitted (A:9, B:3). Verification phase subjects from the PAPR study (**A**) and validation phase subjects from the TREETOP study (**B**) were stratified into higher- (gold) and lower-risk (blue) groups defined by the proteomic biomarker threshold −1.37.

Neonates delivered to subjects with proteomic biomarker scores at or above the threshold had longer hospital stay than those below in both PAPR (*p*-value <0.001) and TREETOP (*p*-value 0.0053) studies (Figure 1).

Those preterm neonates with stays >10 days or perinatal mortality were increased by close to 3-fold at or above the threshold (Table 2, *p*-value < 0.001).

**Figure 2:**
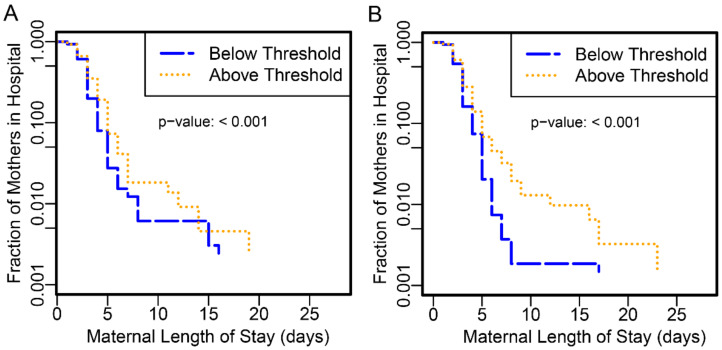
Kaplan–Meier curve of maternal length of stay in days. The fraction of mothers remaining in the hospital is plotted as a function of the length of hospital stay in days. Women without recorded hospital stays were omitted (**A**:3, **B**:0). Verification phase subjects from the PAPR study (**A**) and validation phase subjects from the TREETOP study (**B**) were stratified into higher- (gold) and lower-risk (blue) groups defined by the proteomic biomarker threshold of −1.37.

Likewise, maternal length of stay was significantly longer amongst those subjects with scores above the proteomic biomarker threshold than below for PAPR (*p*-value < 0.001) and TREETOP (*p*-value < 0.001) (Figure 2).

**Figure 3:**
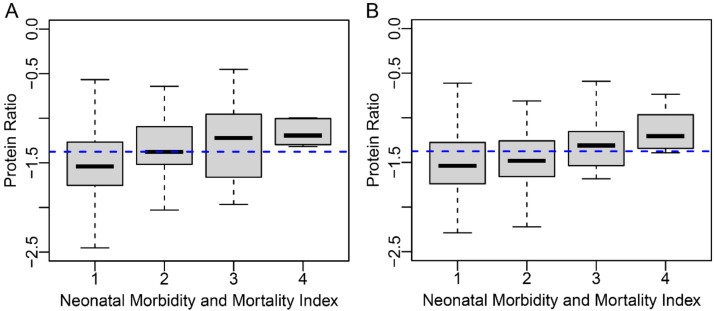
Distribution of all subjects by test score and NMI index. NMI distribution by proteomic biomarker in verification phase subjects from the PAPR study (**A**) and in validation phase subjects from the TREETOP study (**B**) are represented by box plots (box, interquartile range; line, mean; whiskers, remaining range of scores to a maximum of 1.5 box widths). The blue dashed line indicates the proteomic biomarker threshold of −1.37.

Maternal length of stay ≥7 days was increased more than 4-fold at this threshold (Table 2, *p* < 0.001). While PAPR reported total maternal stay only, TREETOP reported antenatal and postnatal stays separately. In TREETOP, subjects at or above the threshold were hospitalized longer, both before and after delivery, than those below the threshold (antepartum *p*-value 0.0013; postpartum *p*-value 0.0027). Antepartum stay ≥5 days was increased 5.3-fold while postpartum stay ≥5 days was increased 2.5-fold. Finally, proteomic biomarker score was associated with levels of severity of NMI (Figure 3, Kendall’s rank correlation *p*-value < 0.001).

Severe NMI (≥3) and mortality were 2.2- and 7.4-fold higher, respectively, in those at or above the proteomic biomarker threshold compared with those below (Table 2, *p*-values 0.0083 and 0.0018, respectively).

## 4. Discussion

Two large studies have been published validating the ability of the ratio of IBP4 to SHBG to risk stratify preterm delivery and associated adverse outcomes [9,16]. The National Academy of Medicine has developed and published guidelines for newly developed molecular tests which advise that such tests be replicated in a second independent study at a specific actionable threshold. Following these guidelines, we assessed an actionable threshold learned in one study and applied to the second in a critical and rigorous manner to show that not only the likelihood of spontaneous preterm delivery is similarly significantly predicted, but also the associated and clinically adverse end points are well predicted and similarly elevated at or above the threshold. A test to predict premature delivery is far more important if it can be shown that it predicts not only premature delivery, but also early premature delivery and the adverse outcomes associated with prematurity, so that interventions can be utilized, developed and tested to decrease the likelihood and/or lessen the severity of the potentially devastating complications of prematurity. Currently, for example, progesterone therapy has been shown to decrease preterm birth [24,25] and, in some studies, improve outcomes [6], but the indications for its use, women with previous spontaneous preterm delivery or short cervix, apply to a small proportion of the pregnancies that ultimately deliver prior to term. In contrast, the two studies of the IBP4/SHBG proteomic biomarker show the ratio’s potential to predict the majority of preterm birth based on tested populations in excess of 1000 subjects, and for predicting associated newborn complications of prematurity as well [9,16].

The primary objective of this research was to demonstrate that statistically significant thresholds of prediction of adverse pregnancy outcomes in PAPR are also significant in the independent TREETOP population. It was of particular interest to test the proteomic biomarker threshold corresponding to a two-fold increased risk of sPTB published in Saade et al. Indeed, in this study the proteomic biomarker threshold of −1.37, corresponding to a risk probability threshold of ~15%, has been clinically validated for predicting elevated sPTB, longer neonatal and maternal length of hospital stays, and more severe neonatal outcomes.

An additional strength of this comparison of the PAPR and TREETOP studies is that while the subpopulations analyzed are both in the same intended use population for the proteomic biomarker, they are notably different on several demographic and baseline characteristics (maternal age, BMI, education, race, prior sPTB, etc.). As well, the eligible PAPR and TREETOP subjects for this study were enrolled at 10 and 14 clinical sites, respectively. All of these factors would provide further confidence that despite these demographic differences and diversity in site enrollment, the same proteomic biomarker threshold identifies pregnancies of increased risk of sPTB and associated adverse outcomes. This is strong evidence of the robust reproducibility and generalizability of the test and the validated risk threshold.

One limitation is that despite the large number of total subjects in the combined studies, the most severe and rare phenotypes analyzed had small numbers of subjects (e.g., eleven delivering with infant mortality and eighteen delivering earlier than 32 weeks).

In conclusion, we have demonstrated consistency and accordance of the proteomic biomarker in two large studies for predicting preterm delivery in a large diverse segment of low-risk pregnant women tested at a time in the second trimester when most women are seen for their anatomic ultrasound. This provides confidence that pregnancies can be robustly risk-stratified by the proteomic biomarker.

## Figures and Tables

**Table 1:** A comparison of the PAPR and TREETOP cohorts.

Variable	PAPR (*n* = 549)	TREETOP (*n* = 847)	*p*-Value
**Maternal age (years)** Mean (SD)	27.47 (5.88)	29.23 (5.36)	<0.001
**Body mass index (kg/m^2^)** Mean (SD)	28.57 (7.77)	27.49 (6.96)	0.010
**Maternal education *n* (%)**			
Unknown	2 (0.36)	3 (0.35)	<0.001
No high school graduation	144 (26.23)	128 (15.11)	
High school graduation or GED	278 (50.64)	381 (44.98)	
College graduation with 4-year degree or higher	125 (22.77)	335 (39.55)	
**Race *n* (%)**			
Black or African American	89 (16.21)	173 (20.43)	<0.001
Other	52 (9.47)	165 (19.48)	
White	408 (74.32)	509 (60.09)	
**Ethnicity *n* (%)**			
Hispanic or Latino	192 (34.97)	335 (39.55)	0.095
Non-Hispanic or Latino	357 (65.03)	510 (60.21)	
Unknown	0 (0.00)	2 (0.24)	
**Gravida *n* (%)**			
Multigravida	389 (70.86)	570 (67.30)	0.174
Primigravida	160 (29.14)	277 (32.70)	
**Prior Full-term Birth *n* (%)**			
First Pregnancy	160 (29.14)	277 (32.70)	0.289
None	76 (13.84)	101 (11.92)	
One or more	313 (57.01)	469 (55.37)	
**Prior sPTB *n* (%)**			
First Pregnancy	160 (29.14)	277 (32.70)	<0.001
None	324 (59.02)	546 (64.46)	
One or more	65 (11.84)	24 (2.83)	
**Delivery *n* (%)**			
miPTB	29 (5.28)	32 (3.78)	0.027
sPTB	37 (6.74)	34 (4.01)	
Term	483 (87.98)	781 (92.21)	
**Fetal Gender *n* (%)**			
Ambiguous	0 (0.00)	1 (0.12)	0.771
Female	282 (51.37)	422 (49.82)	
Male	267 (48.63)	424 (50.06)	
**Neonatal morbidity and mortality index *n* (%)**			
1	482 (87.80)	767 (90.55)	0.083
2	50 (9.11)	55 (6.49)	
3	10 (1.82)	21 (2.48)	
4	7 (1.28)	4 (0.47)	

*p*-value (Fisher’s exact test or Welch’s t-test) is provided for statistical comparisons.

**Table 2:** Comparisons of maternal and neonatal outcomes in the combined PAPR and TREETOP populations at or above versus below the threshold of −1.37.

Threshold	NMI = 3 *n* (%)	NMI = 4 *n* (%)	Maternal Length of Hospital Stay ≥7 Days *n* (%)	Neonatal Length of Hospital stay >10 Days or Mortality, PTB < 37, *n* (%)	Neonatal Length of Hospital stay >10 Days or Mortality, PTB < 35, *n* (%)
Below (negative test)	18 (2.1%)	2 (0.2%)	9 (1.0%)	21 (2.4%)	17 (2.0%)
At or above (positive test)	24 (4.5%)	9 (1.7%)	23 (4.4%)	34 (6.4%)	29 (5.5%)
*p*-value	0.0083	0.0018	<0.001	<0.001	<0.001
Fold change	2.2	7.4	4.2	2.7	2.8

NMI = neonatal morbidity and mortality index. Counts included all infants (term or preterm) with NMI = 3 (severe morbidity) or 4 (neonatal mortality). Likewise, any mother with record of hospital stay ≥7 days was included, regardless of timing of delivery. Lastly, infants with hospital stays >10 days or perinatal mortality that delivered preterm (<37 and <35, respectively), either spontaneous or iatrogenic, were tallied.

## Data Availability

Data supporting the results presented here can be requested at data-sharing@seraprognostics.com. Data will not be made publicly available, or in any format, that may violate a subject’s right to privacy. For example, dating information or identifiers that would allow data to be integrated, thereby enabling the potential identification of study subjects, are protected.
